# Personal

**Published:** 1893-11

**Authors:** 


### Personal.

DR. ROSWELL PARK, of Buffalo, is lying ill of diphtheria at the Kimberly cottage of the Buffalo General Hospital. He contracted the disease in operating on two children of Dr. V. Mott Pierce. It is believed by his physicians that Dr. Park will recover and, indeed, that he has passed already the principal dangers of the malady, for which his many friends will be gratefully thankful.

